# Application prospect of speckle tracking echocardiography in pacemaker implantation

**DOI:** 10.3389/fcvm.2024.1484520

**Published:** 2025-01-03

**Authors:** Nan Xu, Xiaoping Cheng, Lei Ren, Quan Yuan

**Affiliations:** ^1^ Department of Cardiology, The First People’s Hospital of Neijiang, Neijiang, China; ^2^ Department of Ultrasonic Medicine, The First People’s Hospital of Neijiang, Neijiang, China

**Keywords:** speckle tracking echocardiography, traditional right ventricular pacing, His bundle pacing and left bundle branch pacing, cardiac resynchronization therapy, implantable cardioverter-defibrillators

## Abstract

More than 1 million permanent pacemakers are implanted worldwide each year, half of which are in patients with high-grade atrioventricular block. Pacemakers provide adequate frequency support in the initial stage, but traditional right ventricular (RV) pacing may lead to or aggravate left ventricular dysfunction and arrhythmia. Several potential risk factors for heart failure and arrhythmias after pacemaker surgery have been identified, but their occurrence remains difficult to predict clinically. Compared with RV pacing, His bundle pacing (HBP) and left bundle branch pacing (LBBP) activate the intrinsic His–Purkinje conduction system and provide physiological activation, but whether HBP and LBBP also cause ventricular mechanical dyssynchrony remains uncertain. The implantation of cardiac resynchronization therapy and implantable cardioverter defibrillator depends on left ventricular ejection fraction (LVEF). LVEF This depends on volume changes and is less reproducible. Speckle tracking echocardiography (STE) is a technique that can accurately quantify the degree and duration of systolic deformation. STE detects changes in myocardial function more sensitively than traditional measures of diastolic and systolic function, including LVEF. Clinicians can evaluate myocardial strain and synchrony based on strain (percent change in segmental length from baseline) and strain rate (strain per unit time). This review and case series investigate the clinical use of speckle tracking echocardiography in pacemaker implantation.

## 1 Introduction

On 8 October 1958, cardiac surgeon Ake Senning implanted the first complete pacemaker, which is considered the actual birth of today's pacemaker therapy (1). With the continuous development of pacemaker technology, the pacemaker was gradually transferred from thoracotomy to transvenous pacemaker (TV-PM). Pacemaker technology reduces mortality in patients at high risk for second-degree type II or third-degree atrioventricular block (AVB). It also improves the prognosis of patients with bradycardia due to other causes such as sick sinus syndrome (2). To pursue better prognosis, the battery, electrode, and stimulation mode of pacemaker have been gradually adjusted and optimized (3). The availability of leadless pacemakers has reduced the risks of lead dislocation, pocket hematoma, lead failure, and local and systemic infections (4). However, there are still some adverse events after pacemaker implantation. Under physiological conditions, the point activity from the sinoatrial node travels down the His bundle through the atrioventricular (AV) node and reaches the right ventricle and left ventricle through the right bundle branch and left bundle branch, respectively (5). The ideal stimulation mode is to make the pacing signal transmit along the physiological pathway to achieve physiological stimulation. Worsening of systolic function has also been observed when ventricular leads are implanted for frequency support at the right ventricular (RV) apex, septum, and right ventricular outflow tract, whether single-chamber or dual-chamber AV pacing (6). Electrical pulses are not conducted through the Purkinje system, and disruption of normal electrical and subsequent mechanical activation of the ventricle results in delayed basal and lateral activation of the left ventricle (7). This results in systolic hypofunction, elevated filling pressures, and ultimately maladaptive cardiac remodeling and leads to the development of clinical heart failure with PICM (8). The incidence of PICM can reach 10%–20% within 3–4 years after pacemaker implantation (9, 10). His bundle pacing (HBP) and left bundle branch pacing (LBBP) have been developed to provide physiological activation by activating the intrinsic His–Purkinje conduction system (11). However, whether HBP and LBBP also cause ventricular mechanical dyssynchrony is uncertain. For cardiac resynchronization therapy (CRT) and implantable cardioverter defibrillator (ICD), the preoperative implantable pointer depends on left ventricular ejection fraction (LVEF) (12, 13), and there are no clear predictors of postoperative treatment and prognosis.

Speckle tracking echocardiography (STE) tracks acoustic scattering (speckle) of the myocardium frame by frame to calculate strain or deformation of the myocardium. Compared with conventional echocardiography, it is more valuable in evaluating systolic function, cardiac synchrony, myocardial fibrosis, and regional strain (14, 15). These indicators should be paid attention to by clinicians when implanting a pacemaker. This article will discuss the application value of STE technique in pacemaker implantation.

## 2 Speckle tracking echocardiography

**Figure 1:**
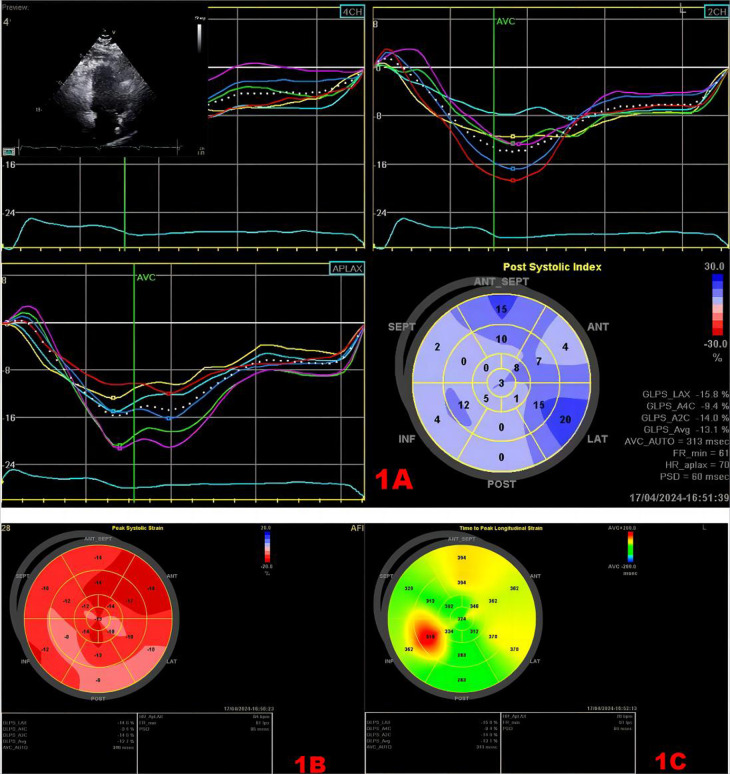
Speckle tracking map. **(A)** Strain time curve and strain peak time plot. Upper left myocardial Doppler 2D measurement image of the region of interest. The middle curve is the strain–time curve. The lower right panel shows the time-to-peak bull's eye plot, and the values in the lower right panel show the strain values, GLS, and longitudinal strain time-to-peak dispersion in each section. **(B)** Bull's eye plot of LV longitudinal strain, with approximately red color representing greater myocardial deformability. **(C)** Bull's eye plot of the time to peak in the left ventricle, the longer the time to peak, the darker the color.

STE is a technique that analyzes motion by tracking “spots” on two-dimensional (2D) or three-dimensional (3D) black-and-white echocardiograms (14). Spots are generated by the reflection and scattering of ultrasound, but they do not exist as true structures. Consecutive frame-by-frame tracking of the spots was performed using the absolute difference sum algorithm. The image processing algorithm tracks user-defined regions of interest (16). Its ultrasound image consists of stable acoustic spots evenly distributed within the myocardium. During the tracking process, small random errors accumulated in speckle pattern detection will lead to inaccurate tracking results, but compared with conventional Doppler ultrasound, there is no angle dependence, and the results are less affected by the examiner (17). STE tracks the distances between frames and points or their spatiotemporal displacements (regional strain velocity vectors) during each cardiac cycle and can distinguish between normal myocardial segmental displacements that occur passively due to myocardial hypertrophy or restriction by adjacent myocardial tissue (18). It provides valuable information on myocardial focal, phasic, and global myocardial strain. Two-dimensional STE (2D-STE) can measure myocardial longitudinal strain (LS) to evaluate cardiac function. Global circumferential strain (GCS) and global radial strain (GRS) were measured in three short-axis and three apical views to obtain global longitudinal strain (GLS) (19). Three-dimensional STE (3D-STE) can automatically measure GCS, GRS, and GLS with only one apical image acquisition (20). These strain indices in STE represent the ratio of the maximum systolic change in myocardial length in each direction to its initial magnitude. During systole, when the myocardium contracts, the length decreases, resulting in strain parameters that are usually expressed as negative values. Lower negative values indicate better ventricular systolic function (21) (Figure 1). Speckle tracking strain can detect the focal changes of myocardial function. In daily use, it has a better value than conventional echocardiography for the early diagnosis of cardiomyopathy, cardiac tumors, and ischemic heart disease (14). Due to bradyarrhythmia implanted with cardiac pacemaker, especially right ventricular apical pacing, electrical stimulation of the pacemaker can cause interventricular and intraventricular dyssynchrony, resulting in structural changes caused by abnormal myocardial perfusion and heart failure (22, 23). STE can be used to assess myocardial synchrony during electrical pacing to predict the risk of heart failure induced by the pacemaker (24). For patients with cardiac dyssynchrony requiring pacemaker resynchronization therapy, STE can evaluate the systolic synchronization of left ventricular segments and identify suitable sites for new activation (25). STE can also be used to guide the left ventricular lead pacing site and the latest activation segment, so as to improve the CRT response rate and prognosis of patients (26).

## 3 Application of speckle tracking technology before pacemaker implantation

### 3.1 Right ventricular pacing

The right ventricular apex is the classic site of pacemaker ventricular lead placement. However, when the proportion of ventricular pacing is more than 40%, right ventricular apical pacing will increase the incidence of heart failure and atrial fibrillation (27). In patients with sinus node dysfunction, dual-chamber pacing can reduce the risk of atrial fibrillation, but it does not improve heart failure and survival (28). This conclusion is consistent with indications for ICD implantation in patients with no indication for cardiac pacing but an LVEF ≤40% (6). The ventricular activation sequence during right ventricular septum pacing (RVSP) is relatively consistent with the physiological law, but the pacing signal cannot be transmitted through the patient's own conduction system, which will lead to conduction delay (29). During RV pacing, the presence of myocardial fibrosis leads to electrical and mechanical dyssynchrony and more severe left ventricular remodeling (30). In addition, local myocardial fibrosis will lead to increased impedance after pacemaker implantation, which will affect the pacing effect and battery life (31, 32). Myocardial scar is an independent predictor of right ventricular pacing-induced heart failure (33).

Therefore, in addition to cardiac structure, the degree of cardiac fibrosis and scarring should also be taken into account before right ventricular pacing. Cardiac magnetic resonance (CMR) late gadolinium enhancement (LGE) is the gold standard for detecting focal and diffuse cardiac chamber fibrosis, but its use is limited by its availability and the use of contrast agents (34). Compared with CMR, STE can be used for evaluation and semi-quantitative analysis without the use of contrast media, and the degree of myocardial fibrosis can be evaluated by measuring myocardial strain (35, 36). In an STE program to identify children and adolescents with focal myocarditis without reduced ejection fraction, patients with sustained reduction in GLS had residual focal myocardial fibrosis on follow-up CMR. The reduction in GLS was consistent with the amount and location of edema on CMR (37). STE can be used to evaluate the degree of myocardial fibrosis and scar before pacemaker implantation, which may have guiding value for the selection of pacing site and pacing parameters.

### 3.2 His bundle pacing and left bundle branch pacing

HBP and LBBP achieve true physiological pacing through their own conduction systems (38). The 2018 ACC/AHA/HRS guidelines for the evaluation and management of patients with bradycardia and cardiac conduction delay state that patients with atrioventricular block and indications for permanent pacemaker implantation with an LVEF between 36% and 50% have a higher risk of cardiac dysfunction. If the proportion of ventricular pacing is predicted to be more than 40%, pacing to maintain physiological ventricular excitation can be selected. The 2023 HRS/APHRS/LAHRS guideline on cardiac physiologic pacing for the avoidance and mitigation of heart failure also pointed out that for patients with LVEF between 36% and 50% and permanent pacing indications, extensive ventricular pacing is expected to be required, and HBP and LBBP are reasonable options to reduce the risk of PICM (8). However, for patients with normal cardiac function and predicted ventricular pacing rate >40%, the guideline does not recommend whether His–Purkinje system pacing (HPSP) is also preferred (39). STE can quantitatively analyze the deformation of the myocardium in the longitudinal, radial, and circumferential directions during the cardiac cycle, and detect myocardial dyskinesia in the early stage. It is a more sensitive indicator of myocardial contraction than LVEF (40). Previous studies have shown that LBBP under the guidance of STE is an optimal pacing mode for patients with preoperative pacemaker dependence and normal LVEF (41). In addition, to ensure LBBP implantation, it is essential to accurately evaluate the cardiac structure, especially the thickness of the basal interventricular septum and the presence or absence of septal scar before operation (38).

### 3.3 Cardiac resynchronization therapy

CRT is a device therapy for heart failure patients with LVEF ≤35% and left ventricular dyssynchrony with QRS duration ≥120 ms (13). STE can evaluate the myocardial dyssynchrony by measuring the maximum time delay between the peak systolic strain of the two segments and the dyssynchrony index of the left ventricle, which is not affected by the measurement Angle and can evaluate the cardiac synchronization more accurately (42, 43). A multicenter prospective study using four speckle tracking methods to assess baseline dyssynchrony demonstrated that circumferential and longitudinal strain predicted response to CRT. This indicates that dyssynchrony measured in STE is associated with prognosis after CRT implantation (44). In addition, studies have shown that mechanical dyssynchrony assessed by STE has high value in predicting responsiveness to CRT. Preoperative STE may be helpful in deciding the indication for CRT, especially if it is necessary to avoid non-response after CRT (45). Abdelfattah et al. also found that STE measurement of maximum interval to lateral delay is a good tool for predicting CRT response before implantation (46).

### 3.4 Implantable cardioverter-defibrillators

ICD is a primary and secondary prevention method for sudden cardiac death in patients with heart failure and structural heart disease (47). It is highly effective in terminating life-threatening ventricular arrhythmias (VA). Current risk prediction used to determine the need for an ICD relies primarily on LVEF (12, 47). But LVEF represents the condition of cardiomyopathy, although not necessarily the tendency to arrhythmia. The meta-analysis showed that LVEF had a sensitivity and specificity of 59% and 78% for significant arrhythmic events, respectively (48). LVEF as a single reliable predictor of VA leading to sudden cardiac arrest (SCA) is questioned (49). STE is an independent predictor of VA in both patients with previous myocardial infarction and patients with non-ischemic heart disease (NIHD), and its predictive value is also confirmed in patients with LVEF >35% (49). In a prospective study of patients with NIHD, STE was more accurate in predicting arrhythmic events than LVEF (50). In studies of patients with heart failure, worsening systolic function as assessed by STE was associated with increased risk for SCA (51). Therefore, some scholars have proposed that STE can be used for arrhythmia risk stratification in patients with ICD, especially in the case of unclear indications. It was considered that the risk of antitachycardia pacing (ATP) or shock with ICD increased at lower GLS, and the hazard ratio (HR) for the first ICD therapy increased by 1.65 for each SD unit of GLS (52). Nikoo et al. also suggested that GLS in patients with hypertrophic cardiomyopathy (HCM) had the highest accuracy in predicting ICD indications in HCM patients, and could be used as a reliable index for early prediction of fatal arrhythmia (53).

## 4 Application of speckle tracking technology after pacemaker implantation

### 4.1 Right ventricular pacing

Right ventricular pacing may cause interventricular and intraventricular dyssynchrony, leading to abnormal myocardial perfusion and endothelial function, poor cardiac output, and clinical manifestations of heart failure and/or arrhythmias (54). This condition is called pacing-induced cardiomyopathy (PICM) (55). Multiple risk factors for PICM include male sex, wider initial and pacing QRS durations, and a higher percentage of right ventricular pacing. However, tools to predict which patients will develop PICM remain limited (55, 56). GLS of left ventricle appeared before the significant change of LVEF (54). It was suggested that GLS measured at 1 month after surgery could predict left ventricular dysfunction 1 year later (57). The lower the baseline GLS measured by STE, the higher the risk of PICM (58). Such patients require more frequent follow-up and may even require His bundle pacing or an upgrade to biventricular pacing (58). Even in the BUDAPEST-CRT Upgrade trial, upgrading to a CRT-D reduced the risk of death from any cause, hospitalization for heart failure, or combined ventricular remodeling among patients with a pacemaker or ICD who had a significant RV pacing burden and a reduced ejection fraction (59, 60). Cardiac synchrony in heart failure patients with previous pacemaker implantation can be used as one of the references for pacemaker upgrading. GLS detected by STE after RV pacing can predict PICM earlier (61). Early detection and intervention of PICM can help to prevent its occurrence (62).

### 4.2 His bundle pacing and left bundle branch pacing

HBP and LBBP activate the intrinsic His–Purkinje conduction system, thereby maintaining ventricular contraction synchrony and are thought to provide physiological activation (63). But whether LBBP also causes ventricular mechanical dyssynchrony remains uncertain (64). STE can measure myocardial strain and quantify left ventricular function and ventricular systolic synchrony. Global and regional ventricular wall function can be analyzed (65). A number of studies have used STE to evaluate even and long-term cardiac conditions in patients after LBBP and RV pacing, providing more clinical evidence for the advantages of LBBP (41, 63, 66). STE used in the evaluation after LBBP implantation may more accurately predict the prognosis of patients and reflect the advantages of LBBP.

### 4.3 Cardiac resynchronization therapy

Patients whose clinical and echocardiographic performance improves after CRT implantation are defined as “CRT responders,” but about 30% who do not improve are called “CRT non-responders” (67, 68). STE allows more strain analysis, including radial strain (myocardial thickening) and circumferential strain (myocardial shortening) in the axial view, and longitudinal strain (myocardial shortening) and transverse strain (myocardial thickening) in the apical view (69). Suffoletto et al. first reported that desynchronization of STE radial strain quantification correlated with CRT response (26). The combination of Doppler imaging (TDI) longitudinal velocity, reverse wall delay, and STE radial strain has added value in predicting CRT response. The Speckle Tracking and Resynchronization (STAR) study is the first prospective, multicenter study to investigate the relationship between speckle tracking strain dyssynchrony and LVEF response and long-term survival after CRT (25). The combined application of speckle tracking radial strain to assess short-term baseline left ventricular dyssynchrony reported by Imanishi et al. was able to more accurately predict long-term outcomes after CRT (70). Delgado et al. showed that STE measurements of left ventricular dyssynchrony, left ventricular lead position, and myocardial scarring predicted long-term survival in patients with ischemic heart failure treated with CRT (71). In addition, it has also been shown that mechanical activation patterns elicited by STE can identify newly activated sites. This parameter can be used to guide the left ventricular lead pacing site and the latest activation segment so as to improve the CRT response rate and prognosis of patients. This can also be a basis for programming pacing parameters after CRT (72–74). Multi-parameter evaluation using STE is expected to improve the prediction of CRT response and may provide more useful information for the selection of CRT patients.

### 4.4 Implantable cardioverter-defibrillators

Shock therapy after ICD implantation is a clinical concern. In the antiarrhythmics versus implantable defibrillators (AVID) trial, 51% received an ICD shock or antitachycardia pacing within the first year. The first arrhythmia treated was ventricular tachycardia (VT) in 63% of cases and ventricular fibrillation (VF) in 13% of cases (75). Shock reduction is particularly important in patients who receive ICD secondary prevention because of the high risk of treatment and the potential for adverse outcomes from ICD shocks (76). Although ICD programming should be adjusted based on prior knowledge of VT/VF, VA is still not predicted (77). Guerra et al. investigated the relationship between GLS and mechanical dispersion in predicting first and subsequent VA events in patients with ICD. It is proposed that lower GLS is associated with higher risk (52). Another study involving 63 patients with ICD also showed that mechanical dispersion and global longitudinal peak strain (GLPS) were independent predictors of appropriate ICD therapy. Measured mechanical dispersion and GLPS help distinguish high-risk patients who benefit from ICD therapy (78).

## 5 Concluding remarks and future perspectives

Although STE is less affected by the angle of examination than TDI, the reproducibility of the obtained values remains a major concern. The clinical application of STE needs to be further confirmed by different researchers in large samples. STE has a more comprehensive detection of the heart than TDI, but it is not as good as MRI in detecting myocardial fibrosis and scar. However, it is undeniable that STE has become a new tool to evaluate regional and global cardiac function, especially mechanical dyssynchrony. These parameters are of great value for the evaluation before pacemaker implantation and the prediction of postoperative adverse events. Speckle tracking can initially evaluate the local myocardial scar before device implantation and guide the electrode implantation site. With the increasing demand for pacemaker implantation, physiological pacing has developed rapidly. STE may provide more clinical evidence for physiological pacing mode. For patients treated with CRT, in the study by Ypenburg et al., radial strain from speckle tracking analysis was used to assess the regional systolic reserve of CRT left ventricular pacing lead, and it was found that the systolic reserve of left ventricular pacing lead region only existed in responders, so it could predict left ventricular reverse remodeling after CRT (79). In HBP and LBBP, in addition to strain analysis, it is helpful to use contrast injection to delineate the tricuspid valve and septal region. The use of a pacing system analyzer that can record His bundle and left bundle electrograms is more helpful for recording and confirming conduction system capture (8). Regardless of the type of pacemaker implanted, early prediction of postoperative adverse events can help adjust the treatment plan in a timely manner and improve the prognosis of patients. Currently, we are witnessing a shift from LVEF to GLS in both research and clinical applications. The evaluation before and after pacemaker implantation still needs to be solved by multimodal imaging. Although there is a lack of large randomized controlled trials to study the choice of pacing mode guided by STE, we now have some clinical data to refine the selection of pacing mode and site by STE. The ideal stimulation mode is pacing signal transduction without reducing myocardial segmental strain.
